# Tunable high-sensitivity sensing detector based on Bulk Dirac semimetal

**DOI:** 10.1039/d2ra05402g

**Published:** 2022-11-14

**Authors:** Xingyu Wang, Jiangchuan Lin, Zhiyang Yan, Zao Yi, Jiaxin Yu, Wei Zhang, Feng Qin, Xianwen Wu, Jianguo Zhang, Pinghui Wu

**Affiliations:** Joint Laboratory for Extreme Conditions Matter Properties, Tianfu Institute of Research and Innovation, Key Laboratory of Testing Technology for Manufacturing Process in Ministry of Education, Southwest University of Science and Technology Mianyang 621010 China yizaomy@swust.edu.cn; Key Laboratory of Science and Technology on Complex Electromagnetic Environment, China Academy of Engineering Physics Mianyang 621900 China fq_soul2000@163.com; School of Chemistry and Chemical Engineering, Jishou University Jishou 416000 China; Department of Physics and Electronic Engineering, Jinzhong University Jinzhong 030619 China; Fujian Provincial Key Laboratory for Advanced Micro-nano Photonics Technology and Devices, Quanzhou Normal University Quanzhou 362000 China

## Abstract

This paper proposes a tunable sensing detector based on Bulk Dirac semimetals (BDS). The bottom-middle-top structure of the detector is a metal-dielectric-Dirac semimetal. The designed detector is simulated in the frequency domain by the finite element method (FEM). And the simulation results indicate that the detector achieves three perfect absorption peaks with absorptivity greater than 99.8% in the range of 2.4–5.2 THz. We analyze the cause of the absorption peak by using random phase approximation theory. The device exhibits good angular insensitivity in different incident angle ranges, and the three absorption peaks can reach 90% absorption rate when the incident angle is in the ranges of 0–60°. And when adjusting the Fermi level of BDS in the ranges of 0.1–0.5 eV, our detector can realize the frequency regulation of the ultra-wide range of 3.90–4.56 THz and realize multi-frequency controllable sensing while maintain the absorption efficiency above 96%. The detector has maximum sensitivity *S* of 238.0 GHz per RIU when the external environment of the refractive index changes from 1.0 to 1.8, and the maximum detection accuracy is 6.5. The device has broad development prospects in the field of space detection and high-sensitivity biosensing detection.

## Abstract 2

We propose a tunable narrow-band sensing detector with an Au-polyimide-Dirac semimetal structure. Simulations show >99.8% absorptivity at 2.4–5.2 THz. The sensor has active tunability, high refractive index sensitivity, angle insensitivity, and good sensing performance.

## Graphical abstract

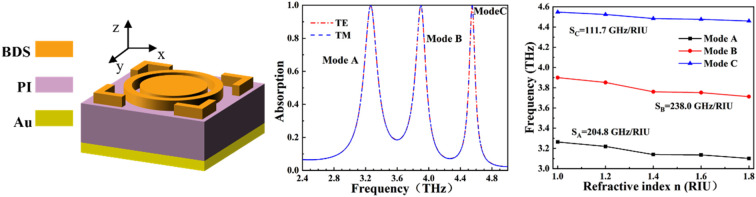

## 1. Introduction

In recent years, the terahertz band has been increasingly studied.^1^ Terahertz waves are also known as ‘terahertz band gaps’ with unique characteristics, which are different from other electromagnetic waves.^2^ It has great potential for development in the fields of biology, security sensing, imaging, photocatalysis and communication^3–6^ since the terahertz band has many unique properties. And the terahertz band is also increasingly used in metamaterials. Metamaterials have electromagnetic characteristics that ordinary natural materials are not available, for instance, negative refractive index, electromagnetically induced transparency, inverse Doppler effect, *etc.*^7^ As a metamaterial, graphene has been extracted since 2004.^8^ Due to its large nonlinear absorption coefficient and refractive index coefficient, as well as its easy dynamic control of optical properties, graphene has been widely used in optical sensing applications.^9–12^ In recent years, researchers have predicted a new topological quantum material based on first-principles theory, three-dimensional Dirac semimetals, also known as bulk Dirac semimetals.^13^ The bulk electrons of this material will form the three-dimensional Dirac cone structure, so BDS is also called ‘3D graphene’,^14^ BDS also falls under the graphene and metamaterials framework.

BDS is a relatively frontier substance in topological semimetals (TSMs). Compared with topological insulators (TIs), TSMs can adjust the interaction of light material under extremely low photon energy, however, optoelectronic detection based on TIs is usually hindered by dark current. This makes TSMs more advantageous in photoelectric detection.^15,16^ BDS can overcome the slow response speed of traditional electronic materials to THz optoelectronics, and some inherent physical constraints.^17^ BDS presents massless relativistic quasiparticles, which exists in the form of a thin film on substrate.^18^ Recently, materials with type-II Dirac semimetals have been found in transition metal dihalides (TMD), which have attracted much attention.^19^ For example, the PtTe_2_ has both a bulk cone and topological protected type-II Dirac surface-state.^20^ PtSe_2_ has unique advantages in the aspect of broadband photon absorption due to the presence of type-II Dirac fermions.^21^ NiTe_2_ and PdTe_2_, which have been predicted to be type-II Dirac semimetals.^22,23^ There is also NiTeSe, an environmentally stable layered Dirac semimetal with a low-energy type-II fermion.^24^

The discovery of several BDS has promoted the development of photoelectric detection detectors. The BDS as metamaterials has metallic properties at terahertz frequencies, and they can act as a detector's ‘Salisbury screen’ to block transmission, so BDS is particularly suitable as an absorbing material in the terahertz band. At present, many detectors designed are not achieve the function of active tuning. Once the model is formed, it is difficult to tune again. It can only absorb electromagnetic waves of a specific frequency, and the absorption performance is inflexible. There is an increasing number of researches on tunable absorbing materials. Devices designed with tunable materials, not only can effectively reduce cost, but also can be flexibly applied in different resonance frequencies. There are many materials with adjustable capabilities. For example, graphene, VO_2_ and Ge_2_Sb_2_Te_5_.^25–28^ These materials have the ability to be regulated, but at the same time have certain limitations. In order to cope with the complex electromagnetic environment, there are higher design requirements for the construction of tunable detectors. BDS can control the wavelength of the surface plasmon resonance, and then the Fermi level of BDS is also adjusted.^29–31^ Absorption detector is composed of BDS, which can realize the active adjustment of operating frequency and absorption rate. Under normal circumstances, there are two ways to adjust the dielectric constant of BDS. One is to dynamically adjust its Fermi level (*E*_F_) by doping the alkaline surface. Because the doping process is difficult to control, it is not suitable for practical applications.^32^ The other is to use the bias voltage dynamically modulates the surface conductivity *σ* of the BDS,^33,34^ thereby changing the permittivity and Fermi level. By manipulating the Fermi level of BDS, the control of absorption at different terahertz frequencies can be achieved. The good controllability of BDS makes it more widely used in tunable detectors.

In general, there are three types of absorption sensors, namely optical sensors, physical sensors, and electrochemical sensors.^35–39^ At present, there are few reports on the application of BDS in optical sensors. In 2017, Wang *et al.* designed a high-performance absorber by exploiting the special absorption properties of BDS metamaterials in the terahertz band.^40^ In 2019, Luo *et al.* constructed a kind of high sensitivity tunable detector based on the BDS, realizing narrow-band absorption and dynamic tunability.^41^ In 2020, Yan *et al.*, designed the ASR structure of Dirac semimetals, which further improved the sensitivity of the device.^42^ Taking advantage of the good properties of BDS, there is still a lot of room for development and great application prospects in the researches of sensor detection. Due to a lot of physical and chemical information is contained in terahertz spectrum, the applications in optical biosensors are also increasing. Optical biosensors use the changes of refractive index to detect biosensing elements, which is an important label-free detection method.^43–45^ The foremost sensing parameters of optical biosensors, for instance, sensitivity, resolution, and detection accuracy, have also been greatly improved.

A tunable high-sensitivity sensing detector based on Bulk Dirac semimetals was proposed in this paper. Three perfect absorption peaks with absorptivity greater than 99.8% were achieved in the range of 2.4–5.2 THz. It realized multi-frequency adjustable sensing detection in ultra-wide range of 3.90–4.56 THz. The absorption performance under various environment with different refractive indexes was studied, and the sensing performance was analyzed. It exhibits good angular insensitivity in a large ranges of incident angle, which can cope with complex electromagnetic environment and is suitable for many fields. In our proposed sensor, the maximum sensitivity *S* reaches 238.0 GHz per RIU when *n* increasing from 1.0 to 1.8. It shows certain significance for the development of biosensors.

## 2. Structure design and theoretical analysis

**Fig. 1:**
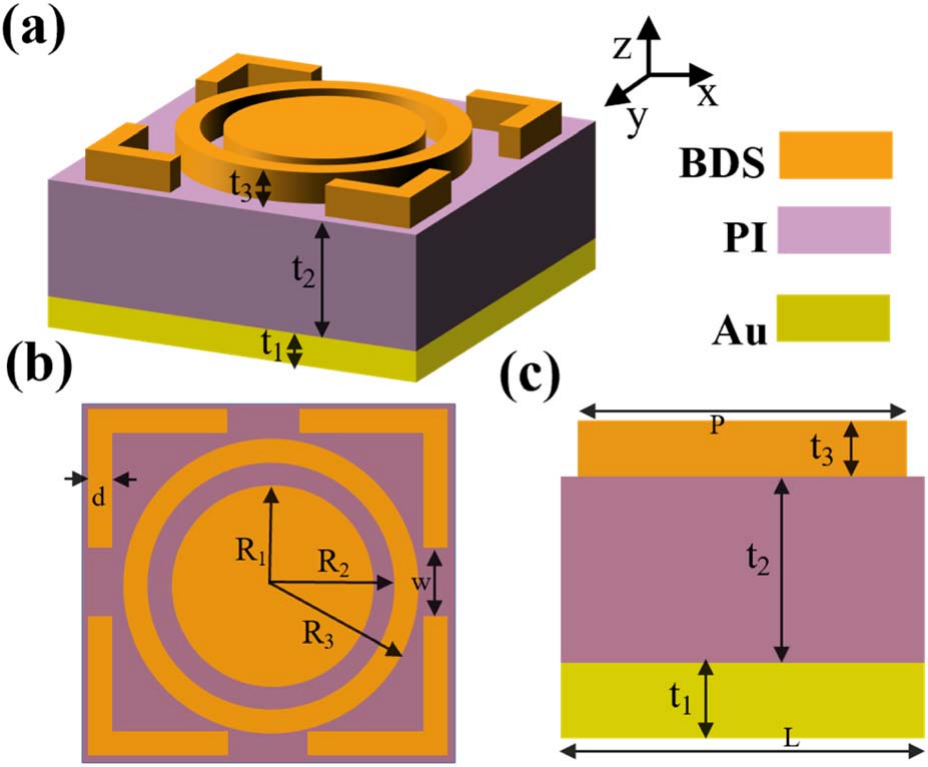
(a) 3D schematic diagram; (b) top view of a detection device; (c) side view of the structure.

Fig. 1(a) is the three-dimensional schematic diagram of the detector we presented. The device is a three-layer spatial construction from bottom to top. The bottom layer is gold, and the thickness *t*_1_ of the gold layer is 0.2 μm. The middle layer is polyimide (PI), with the dielectric constant 3.9 and the thickness *t*_2_ = 6 μm. And the thickness of the top Dirac semimetal layer is *t*_3_ = 1.6 μm. Fig. 1(b) is the overhead view of the detector construction. The top Dirac semi-metal structure consists of three parts, the first part is the middle round pie structure, whose radius *R*_1_ = 4.2 μm. The second part is the ring structure, with the inner radius *R*_2_ = 5.2 μm and the outer radius *R*_3_ = 7.2 μm. The third part is the outermost square ring structure. A rectangular strip with width of *d* = 4 μm and length of *w* = 1.2 μm is removed from the four sides of the square ring structure. The length of the outermost structure is *P* = 17 μm, the width *d* = 4 μm, and the side lengths of the gold layer and polyimide are both *L* = 20 μm.

**Fig. 2:**
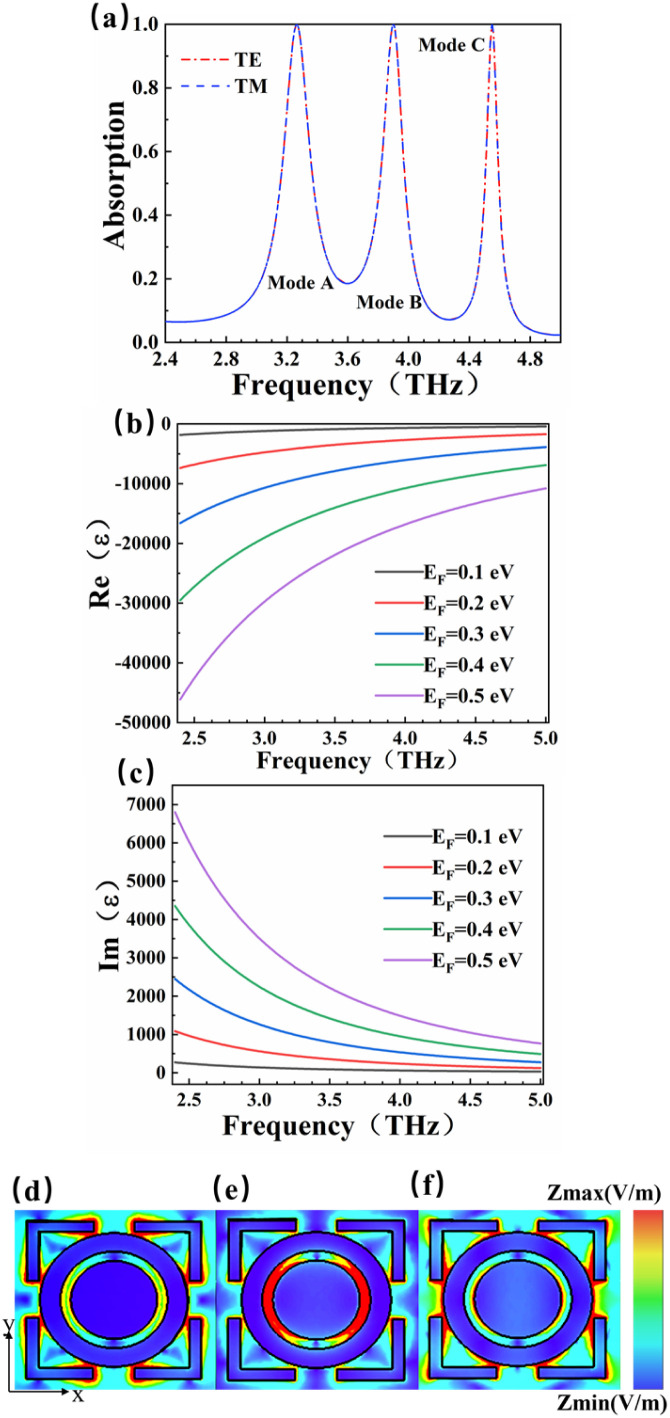
(a) Absorption spectra of detectors in TE and TM modes; (b) real part as a function of Fermi level; (c) imaginary part as a function of Fermi level; (d–f) electric field distribution of resonant frequency of modes A, B, C in the *Z*-direction.

The absorption characteristics of the detector were numerically simulated and analyzed by CST Microwave Studio software.^46,47^ The frequency domain solver was chosen as the solution type. In terms of boundary condition settings, the *x* and *y* directions were set as periodic boundary conditions, and the *z* direction was set as open boundary conditions. In the CST microwave studio, the frequency range studied was 2.4–5.0 THz, and the simulation results are shown in Fig. 2. We got absorption spectra under TE and TM polarization modes respectively. It can be found from Fig. 2(a) that the absorption spectra of the detector under two polarization modes coincide well with each other. This is due to the central symmetry of the detector structure. The results indicate that the absorption spectrum does not correlation with the polarization direction at the incident. There are three resonance peaks with high absorption rate exceeding 99% for different polarization directions. In the TE mode, the resonant frequencies that generate the formant peaks are 3.26 THz, 3.90 THz, and 4.55 THz, while the corresponding absorption rates of the three resonance peaks are 99.97%, 99.99% and 99.83%. (The following studies are all conducted in the TE mode).

Perfect absorption in a particular terahertz band requires zero reflection and zero transmission of the incident object, thus the energy of the electromagnetic wave is completely absorbed. The absorptivity of the absorber structure is usually defined as *A* = 1 − *R* − *T*.^48,49^ In the formula, *A* is the absorption rate, *R* is the reflectivity, and *T* is the transmissivity. The metal layer can achieve zero transmission of the terahertz band. Only reflectance and absorption need to be considered. Therefore, the absorption rate can be simplified as *A* = 1 − *R*.^50,51^ The dynamic conductivity can be obtained in the long-wave limit by using the Kubo formula in the random-phase approximation theory (RPA). The dynamic conductivity of BDS can be defined as:^52^1
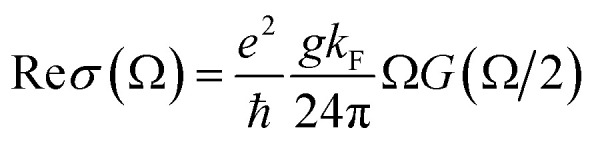
2
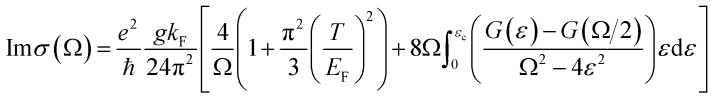


This is the longitudinal dynamic conductivity of the Dirac. *G*(*E*) = *n*(−*E*) − *n*(*E*), *n*(*E*) represents the Fermi distribution function, *K*_F_ = *E*_F_/ℏ*V*_F_ is the Fermi momentum, *V*_F_ is the Fermi velocity, Ω = ℏ*ω*/*E*_F_, *ε* = *E*/*E*_F_, where *E*_F_ is the Fermi level, *V*_F_ is the Fermi energy velocity. In addition, *E*_c_ is the cut-off level, and *E*_c_ < 3 is a necessary condition to maintain the linearity of the Dirac spectrum. According to eqn (1) and (2), where Ω = ℏ*ω*/*E*_F_ is a function of normalized frequency. Similarly, we can calculate a similar function expression as in RPA At the low-temperature limit (*T* ≪ *E*_F_).^53,54^3
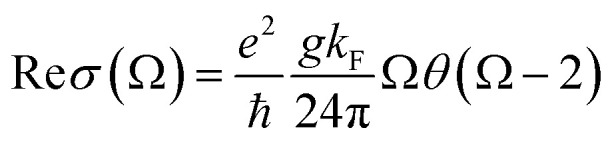
4
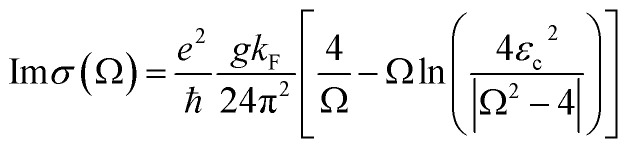


The dynamic conductivity of BDS includes inter-band conductivity and intra-band conductivity, and the dielectric constant *ε* of BDS can be written as:^55^

5 *ε* = *ε*_b_ + i*σ*/*ωε*_0_

In the formula, *ε*_b_ is the effective background permittivity of the BDS material. *ε*_0_ is the vacuum dielectric constant, *ε*_0_ ≈ 8.85 × 10^−12^ F m^−1^.^56^ The formula can be used to simulate and calculate the changes of the dielectric constant of BDS with the frequency of the light wave. The variation of Re(*ε*) and Im(*ε*) with Fermi level is shown in Fig. 2(b) and (c). The real part of the BDS conductivity also arises from the interband transitions and is responsible for the optical absorption.^52^ The imaginary part of the BDS conductivity is dependent on the cut-off level. We calculated the variation of the dielectric constants Re(*ε*) and Im(*ε*) with frequency light waves in the ranges of 0.1–0.5 eV. Based on the localized surface plasmon resonance theory, when electromagnetic waves appear on the BDS surface, the interface between BDS and polyimide will excite localized surface plasmon resonances. In order to further explore the mechanism of generating formant peaks, we set up three field monitors in the CST microwave studio and obtained the electric field intensity diagrams of resonant frequencies in different modes in Fig. 2. This is the scalar distribution of the detector surface electromagnetism in the *z* direction. And it represents the electric field strength of the three modes at the same horizontal cross section. The closer the color is to red, the stronger the magnetic field. The central resonance frequency of Fig. 2(d) is 3.26 THz, it can be found that the tail of the square ring structure has the darkest color and the largest field strength, which is the main region of the local surface plasmon resonance of mode A. The local surface plasmon resonance of this region determines that the detection absorber can absorb perfectly at the incident of 3.26 THz, and the absorption rate reaches 99.99%. In Fig. 2(e), the center frequency is 3.90 THz, and the field strength is the largest at the intersection of the ring and the round cake, which is the main region of the local surface plasmon resonance of mode B. In Fig. 2(f), the center frequency is 4.55 THz, the area around the ring is the reddest and the field is the strongest, which is the main region of the local surface plasmon resonance of mode C. The three resonance peaks are caused by the localized surface plasmon resonance in different regions.

## 3. Results and discussions

**Fig. 3:**
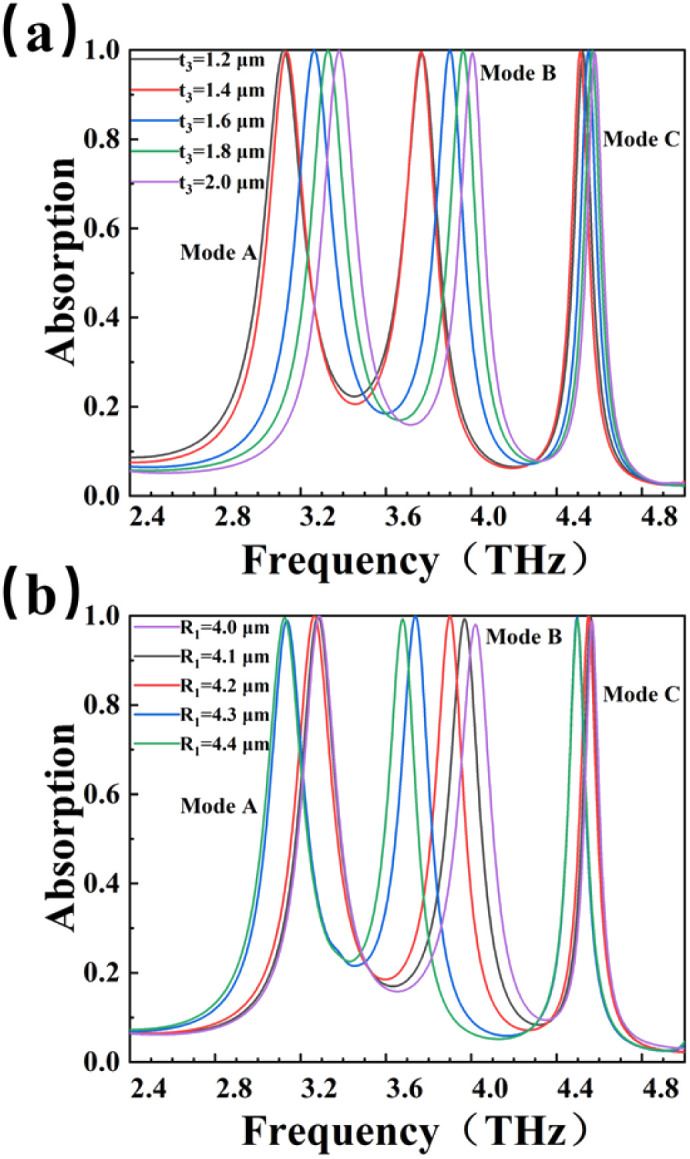
Effect of structural parameters on absorption performance (a) different thickness *t*_3_ of Dirac metal layer; (b) different circle radius *R*_1_.

We study the influence of two structural parameters on sensor performance in order to optimize the parameters. Fig. 3(a) reveals the effect of the height *t*_3_. It can be found that as the height of the BDS layer increases, the formants are blue shifted. The phenomenon for the blue shift can be explained by the equivalent circuit principle. In the BDS structure, the cavity between the parts of the BDS can be regarded as the equivalent capacitance. According to capacitance formula: *C* = *S*/*d*, with the increase of the thickness *t*_3_. The plate area *S* of the equivalent capacitance increases continuously, and the distance *d* between cavities remains unchanged. This leads to an increase in the total amount of equivalent capacitance. The larger the equivalent capacitance, the smaller the impedance, and the higher the frequency of passage. The resonant frequency variation of mode A and mode B is relatively large, while the resonant frequency variation of mode C is relatively small. However, these three modes all maintain high absorption efficiency, and the absorption peaks are above 99%. By changing the height of the BDS layer, the resonant frequency can be shifted in a certain band range. This makes it detect more wide frequency. Fig. 3(b) studies the effect of the radius *R*_1_ of the center circle of BDS. As the radius of the central circle increases, the resonance peaks appear shifts and the shifts of mode B absorption peak is more obvious. By changing the radius of the central circle, we can provide ideas for the regulation of mode B. Moreover, the three modes maintain high absorption efficiency.

**Fig. 4:**
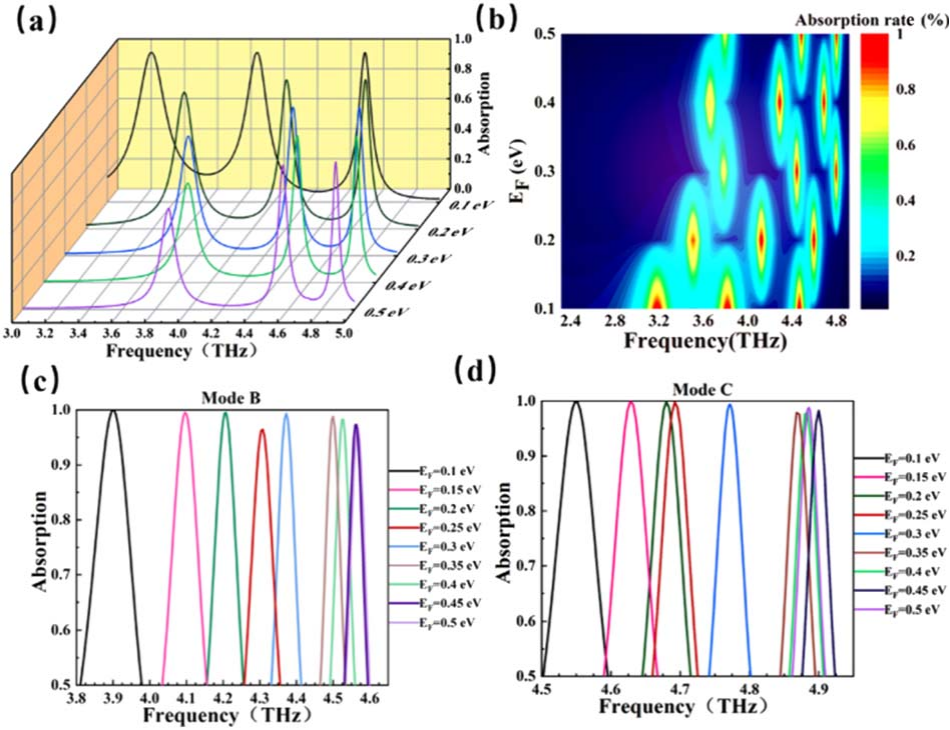
(a) Absorption properties of changing Fermi level; (b) shift of frequency center; (c) frequency control range of mode B (d) frequency control range of mode C.

We know that the Fermi level of BDS can be dynamically regulated, and then the resonant frequency of the device can be regulated. According to this principle, we changed the Fermi level of BDS to discuss the effect on device performance. As shown in Fig. 4(a), the *E*_F_ regulation range of BDS is 0.1–0.5 eV, with the interval of 0.1 eV. It can be found that with the increase of *E*_F_, the absorption curve is blue shifted, and the resonance frequencies of modes A, B, and C all shift. As the *E*_F_ of BDS increases, the real part of permittivity of BDS decreases, the imaginary part of permittivity of BDS increases, thus results in the blue shift of the resonance frequency of the proposed absorber. With the increases of *E*_F_, the resonant frequency of mode A shifts from 3.26 THz to 3.88 THz, and the absorption rate decreases greatly, from 99.97% to 67.74%. And the resonant frequency of mode B shifts from 3.90 THz to 4.56 THz, the absorption rate decreases from 99.99% to 96.62%, and the absorption rate remains above 96%. The resonance frequency of mode C increases from 4.55 THz to 4.88 THz, and the absorption rate decreases from 99.77% to 98.78%. It can be clearly seen from Fig. 4(b) that with the increases of *E*_F_, the center of the resonance frequencies of the three modes all shifted. Each circle represents a peak, and the central region of the bright red color is the location of the formant. And both mode B and mode C maintain high absorption rates. Fig. 4(c) and (d) illustrate the good controllability of mode B and mode C for the resonant frequency. Here, we simulated and calculated eight Fermi levels to prove it. The results are shown below. When adjusting the Fermi level of BDS in the ranges of 0.1–0.5 eV, our detector can realize the frequency regulation of the ultra-wide range of 3.90–4.56 THz, the adjustment of the frequency range can be as high as 0.66 THZ and realize multi-frequency controllable sensing while maintain the absorption efficiency above 96%.

To further illustrate the advantages of resonance frequency controllability of the detector, we have made a comparison of the resonance frequency ranges of some of the detectors that have been reported, as shown in Table 1. These reported absorbing devices are all Dirac semimetals as structural materials, which shows that the device proposed in this paper have greatly improved in the control ranges.^57–60^

**Table 1:** Compare the control range of some detectors

Fermi level modulation ranges	Absorption frequency interval (THz)	Absorption frequency range (THz)	Ref.
50–80 meV	1.38–1.39	0.01	57
30–70 meV	1.08–1.12	0.04	58
45–75 meV	1.72–1.90	0.18	59
0.11–0.15 eV	2.90–3.35	0.35	60
0.1–0.5 eV	3.90–4.56	0.668	Proposed

**Fig. 5:**
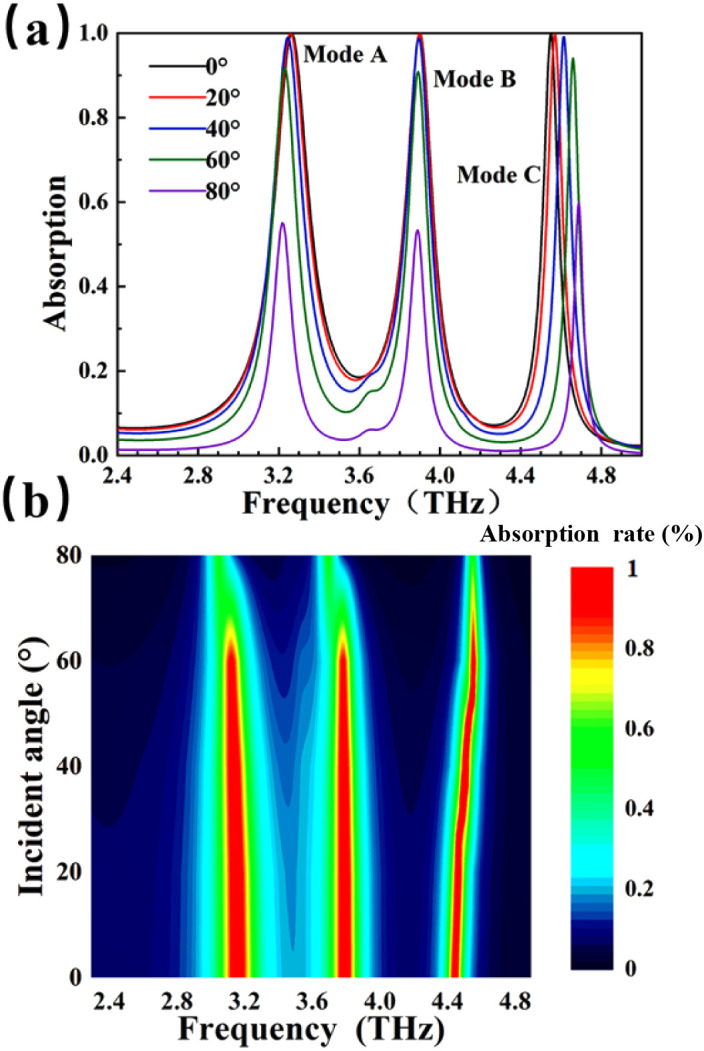
Changing different incident angles. (a) Absorption properties at different incident angles; (b) relationship between absorptivity and incident angle.

During the actual use of the detection device, in order to cope with a more complex electromagnetic environment, the performance requirements of the detector will also be higher.^61–63^ We explored the effect of electromagnetic waves incident from different angles on the absorption spectra of detectors. The ranges of the electromagnetic wave angle are 0°–80°. In Fig. 5(a), when the frequency ranges are 2.4–4.8 THz, the absorption rate of modes A, B and C can be kept above 90% in the incident ranges of 0–60° under the TE mode, and the absorption intensity does not change significantly due to the highly symmetrical structure of the device. When the incidence Angle exceeds 60°, the absorption efficiency starts to weaken gradually. This is due to the gradual weakening of the local surface plasmon resonance and the vertical component of the electromagnetic field decreases. Fig. 5(b) shows the absorptivity *versus* incident angle. The environmental adaptability of the detector is particularly strong, and it exhibits strong angle insensitivity, which makes it have a wider range of detection applications.

**Fig. 6:**
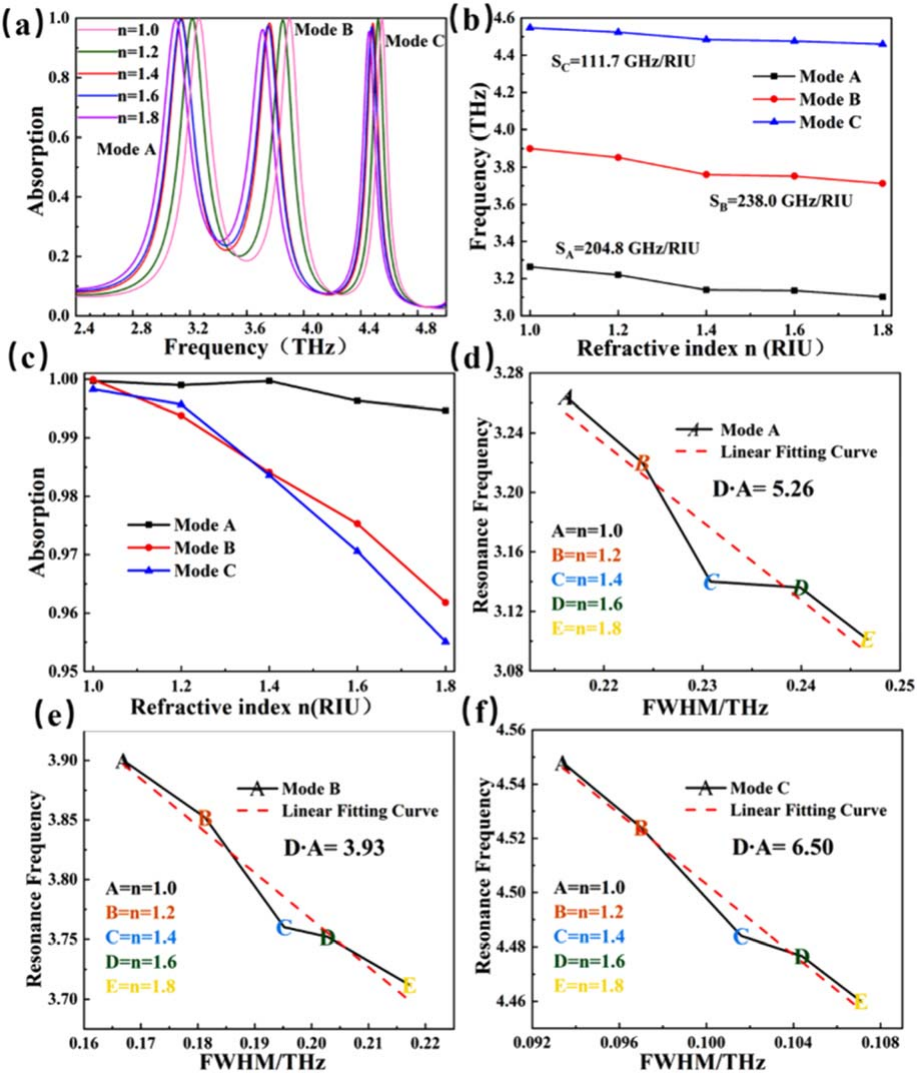
(a) The influence of different refractive index environments on detector performance; (b) influence of different refractive index environments on detector resonance center; (c) effects of different refractive index environments on the absorption intensity of detectors; (d–f) detection accuracy of modes A, B, C.

Environmental refractive index will affect the performance of the sensor. In the case of other structure parameters unchanged, we changed the environmental refractive index, and the effect of the actual situation on the performance of the detector was simulated. As shown in Fig. 6(a), the refractive index changes in the ranges of 1.0–1.8, and the increment interval of refractive index is 0.2. The refractive index is very consistent with the refractive index of conventional materials. In the process of increasing the refractive index value, all the absorption curves appear red-shift. But the resonance intensity does not change significantly. As *n* increases, the resonant frequencies of modes A, B, and C also shift. The offset of the resonance mode can be attentioned as an indicator of the inspection item of the detector. Here we define a quantity that describe the sensitivity of the resonance peak, the sensitivity *S* can be obtained from:^64,65^6
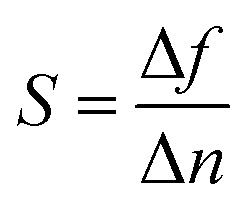


Its unit of S is (GHz per RIU) and it represents the angular coefficient of the linear fit function of Fig. 6(b), where Δ*f* is the variation of the resonant frequency, and Δ*n* is the variation of the external refractive index. The sensitivity is the core index to evaluate the performance of the sensor. Sensitivity is particularly important when designing optical biosensor. From Fig. 6(b), it can be found that the sensitivity of mode A is 204.8 GHz per RIU, the sensitivity of mode B is 238.0 GHz per RIU, and the sensitivity of mode C is 111.7 GHz per RIU. Mode B has the highest sensitivity. Both modes A and B have relatively strong detection capabilities. Fig. 6(c) depicts the offset of absorption intensity of modes A, B, and C. As the refractive index increases, the absorption intensities of mode A maintain high intensity and high stability, and the absorptivity is always above 99.47%, while modes B and mode C change more obviously, and the absorption rate can still be extremely high, maintaining above 95%.

To further illustrate the good sensitivity of the detector, we have made a comparison on the sensitivity of our detector with some of reported detectors, as shown in Table 2.^66–70^ It can be found that the detector designed by us is not only much more sensitive, but also has a larger range of frequency detection. This makes the detector have broader application prospects in high sensitivity biosensing detection. The detection accuracy (*D* × *A*) of the sensing detector, one of the most important parameters of the detector, also known as the signal-to-noise ratio, is defined as:^71^7
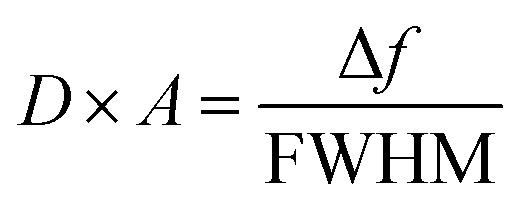
where FWHM (Full Width at Half Maximum) represents the spectral width corresponding to the 50% absorption rate of the absorption curve. Generally, the higher the detection accuracy of the sensor, the better the resolution of the spectral lines.^72,73^Fig. 6(d–f) shows the detection accuracy of the three modes, with *D* × *A* = 5.26 for mode A, 3.93 for mode B and 6.50 for mode C. It is no doubt that mode C has the highest detection accuracy and can detect information more intuitively.

**Table 2:** Compare the sensitivity of some detection devices

Operating band (THz)	Number of peaks	Tunable material	*S* (GHz per RIU)	Ref.
0.2–0.4	1	SSR	24.9	66
0.0–4.0	>3	Graphene	66	67
1.0–2.8	>3	BDS	122.0	68
0.1–0.8	3	Metal	139.2	69
1.0–2.4	3	BDS	152.5	70
2.4–5.0	3	BDS	238.0	Proposed

In the actual use of the detector, the performance of the detector will also be evaluated by the figure of merit (FOM). Therefore, when exploring sensitivity, we should also pay attention to the FOM, the FOM is written as:^74,75^8
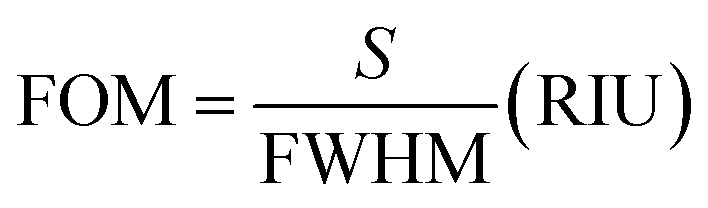


**Fig. 7:**
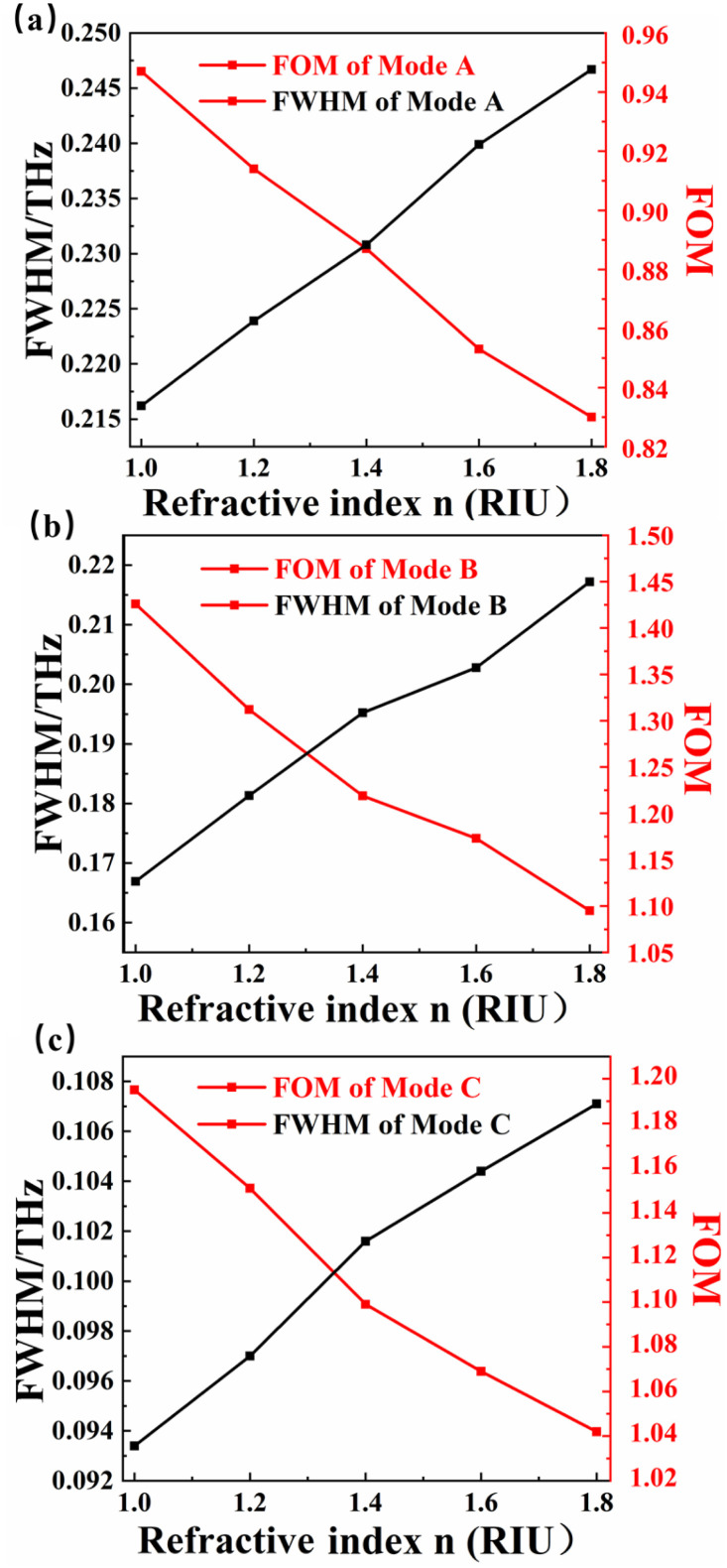
(a–c) Comparison of FWHM and FOM of mode A, B, C.

FOM is also one of the core parameters for evaluating sensors. The higher the FOM, the higher the resolution of the sensor.^76–78^Fig. 7(a) is a contrast of the FWHM and FOM of mode A. As the refractive index of the external environment increases linearly from 1.0–1.8, the half-peak full wave of mode A increases linearly, and the corresponding FOM decreases linearly. At *n* = 1.0, the FWHM reaches the maximum value of 0.246 (THz). Fig. 7(b) compares the FWHM of mode B with the FOM of mode B. The situation is similar to that of mode A. When *n* = 1.0, the FOM of mode B reaches the maximum value of 1.42 (RIU). Fig. 7(c) is a comparison of FWHM and FOM of mode B, and the results are similar to those of mode A. A trend can be seen from the figure, with the changes of refractive index, there is a linear negative correlation for FOM and a linear positive correlation for FWHM. All three modes are more suitable for terahertz wave detection in low index materials. A sensor with good comprehensive performance should not only consider the parameters *S*, FOM and *D* × *A*, but also the detection range, detection environment and so on. The sensor detection device we designed have superior performance in this regard.

## 4. Conclusions

To sum up, we propose a perfect absorption sensing detector based on Dirac semimetals. By changing the Fermi level, the absorption frequency can be tuned in a wide range, and the tuning range reaches 0.668 THz. Three perfect absorption peaks with absorption rates greater than 99.8% realize multi-frequency controllable sensing detection. In the ranges of 0–60°, three absorption peaks can reach 90% absorption, showing strong angular insensitivity. At the same time, our detector also has high refractive index sensitivity, the maximum sensitivity can reach 238.0 GHz per RIU, and the detection accuracy is large, which makes our detector form various types and apply in different fields, especially in the application of biosensor detection prospects.

## Conflicts of interest

There are no conflicts to declare.

## Supplementary Material
